# Effects of linagliptin vs glimepiride on cognitive performance in type 2 diabetes: results of the randomised double-blind, active-controlled CAROLINA-COGNITION study

**DOI:** 10.1007/s00125-021-05393-8

**Published:** 2021-02-09

**Authors:** Geert Jan Biessels, Chloë Verhagen, Jolien Janssen, Esther van den Berg, Gudrun Wallenstein, Bernard Zinman, Mark A. Espeland, Odd Erik Johansen

**Affiliations:** 1 Department of Neurology, UMC Utrecht Brain Center, University Medical Center Utrecht, Utrecht, the Netherlands; 2 Department of Neurology, Erasmus MC - University Medical Center, Rotterdam, the Netherlands; 3 Biostatistics and Data Sciences, Boehringer Ingelheim, Ingelheim, Germany; 4 Lunenfeld-Tanenbaum Research Institute, Mount Sinai Hospital, University of Toronto, Toronto, Canada; 5 Department of Biostatistics and Data Science, Wake Forest School of Medicine, Winston-Salem, NC USA; 6 Therapeutic Area Cardiometabolism, Boehringer Ingelheim, Asker, Norway

**Keywords:** Cardiovascular disease, Cognitive decline, DPP-4 inhibitors, Sulfonylureas, Type 2 diabetes

## Abstract

**Aims/hypothesis:**

Type 2 diabetes, particularly with concomitant CVD, is associated with an increased risk of cognitive impairment. We assessed the effect on accelerated cognitive decline (ACD) of the DPP-4 inhibitor linagliptin vs the sulfonylurea glimepiride in individuals with type 2 diabetes.

**Methods:**

The CAROLINA-COGNITION study was part of the randomised, double-blind, active-controlled CAROLINA trial that evaluated the cardiovascular safety of linagliptin vs glimepiride in individuals with age ≥40 and ≤85 years and HbA_1c_ 48–69 mmol/mol (6.5–8.5%) receiving standard care, excluding insulin therapy. Participants were randomised 1:1 using an interactive telephone- and web-based system and treatment assignment was determined by a computer-generated random sequence with stratification by center. The primary cognitive outcome was occurrence of ACD at end of follow-up, defined as a regression-based index score ≤16th percentile on either the Mini-Mental State Examination (MMSE) or a composite measure of attention and executive functioning, in participants with a baseline MMSE score ≥24. Prespecified additional analyses included effects on ACD at week 160, in subgroups (sex, age, race, ethnicity, depressive symptoms, cardiovascular risk, duration of type 2 diabetes, albuminuria), and absolute changes in cognitive performance. Participants, caregivers, and people involved in measurements, examinations or adjudication, were all masked to treatment assignment.

**Results:**

Of 6033 participants recruited from hospital and primary care sites, 3163 (38.0% female, mean age/diabetes duration 64/7.6 years, MMSE score 28.5, HbA_1c_ 54 mmol/mol [7.1%]) represent the CAROLINA-COGNITION cohort. Over median 6.1 years, ACD occurred in 27.8% (449/1618, linagliptin) vs 27.6% (426/1545, glimepiride), OR 1.01 (95% CI 0.86, 1.18). Also, no differences in ACD were observed at week 160 (OR 1.07 [0.91, 1.25]), between treatments across subgroups, or for absolute cognitive changes.

**Conclusions/interpretation:**

In a large, international outcome trial in people with relatively early type 2 diabetes at elevated cardiovascular risk, no difference in risk for ACD was observed between linagliptin and glimepiride over 6.1 years.

**Funding:**

This study was sponsored by Boehringer Ingelheim.

**Trial registration:**

ClinicalTrials.gov NCT01243424.

**Graphical abstract:**

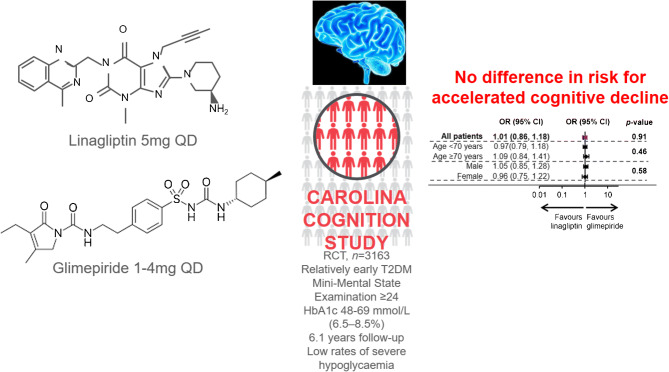

**Supplementary Information:**

The online version contains peer-reviewed but unedited supplementary material available at 10.1007/s00125-021-05393-8.

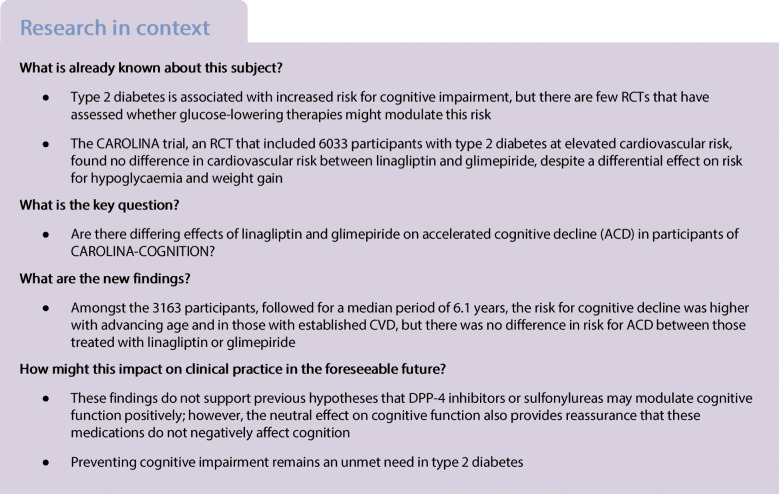



## Introduction

Type 2 diabetes is associated with increased risk for cognitive impairment, both mild cognitive impairment (MCI) and dementia [1], in particular in those patients with coexisting CVD [2]. More subtle cognitive changes, also referred to as cognitive decrements, can be encountered in patients with type 2 diabetes of all age groups, usually with only modest deterioration over time [1, 3]. Yet, accelerated cognitive decline (ACD), which may evolve to MCI or dementia, predominantly occurs in higher numbers in the older age groups [1, 3–5]. This is worrying, since in people 60 years or older with type 2 diabetes, the annual risk of dementia has been observed at 2.6% [6], and older people will constitute the majority of individuals with type 2 diabetes in the USA and most other developed countries in the next decades, with type 2 diabetes currently estimated to affect 19.3% of people 65 years or older worldwide [7]. Cognitive dysfunction in type 2 diabetes, in particular severe forms, is an independent predictor for adverse clinical outcomes such as hypoglycaemia, cardiovascular events and death [1, 8].

The pathophysiology of cognitive decline in type 2 diabetes is complex, likely sharing pathways with conditions such as Alzheimer’s disease and vascular dementia [1, 9, 10]. The hallmark of type 2 diabetes, hyperglycaemia, has also been associated with incident dementia, both in people with and without type 2 diabetes [11], in the latter including a possible non-linearity where both high and low HbA_1c_ levels are linked to poorer cognitive functioning [12]. However, interventions aimed at achieving near-normoglycaemia have not been shown to reduce the risk for cognitive decline [13]. Given the association between type 2 diabetes and MCI and dementia, it is therefore of interest to assess whether the non-glycaemic effects of specific glucose-lowering medications affect cognitive function. This is also of particular relevance in light of some conflicting results on cognitive functioning reported with the use of metformin [5, 14, 15] and insulin [16, 17].

Incretin-based glucose-lowering medications have emerged as a potential therapeutic agent for Alzheimer’s disease [18]. The underlying hypothesis of potential benefit is related to the non-glycaemic effects of this class of drug, involving a variety of direct or indirect targets in the brain for glucagon-like peptide-1 (GLP-1) receptor agonists and dipeptidyl peptidase (DPP)-4 inhibitors, including neuronal cells, different glial cells and neuronal precursor cells [19]. Animal data show that DPP-4 inhibitors may suppress blood–brain barrier disruption and attenuate cerebral oxidative stress or neuroinflammation, and may reduce brain damage following a stroke [19]. The findings of a recent, adequately powered, clinical study comparing effects on cognitive function of dulaglutide vs placebo also support this notion: a modest beneficial effect on a cognitive outcome, defined as first occurrence of a follow-up score on the Montreal Cognitive Assessment or Digit Symbol Substitution Test that was 1.5 SD or more below the baseline mean score in the participant’s country, was seen in an exploratory analysis [20].

Another widely used class of glucose-lowering medication investigated for effects on the cerebrovascular system are sulfonylureas, which also have been suggested to have potential neuron protective effects in various neurological disorders [21, 22]. However, its association with increased risk of hypoglycaemia could offset such benefits, as hypoglycaemia may increase the risk of cognitive impairment in type 2 diabetes [23, 24]. Preclinical studies suggest that sulfonylureas reduce brain infarct volumes and oedema and haemorrhagic conversion, and improve outcomes in rodent models of ischaemic stroke, mainly related to involvement of the sulfonylurea receptor 1 [22]. To date, however, there are no robust clinical data to support a benefit of either DPP-4 inhibitors or sulfonylureas on cognitive functioning [5, 13, 25, 26], (see also literature review conducted up to 10 July 2020, electronic supplementary material [ESM] Table 1), and no direct comparisons have been performed.

The CARdiovascular Outcome study of LINAgliptin versus glimepiride in type 2 diabetes (CAROLINA) was the first head-to-head active-controlled outcome trial of these two classes of medication, and involved 6033 participants with relatively early type 2 diabetes with cardiovascular safety as primary outcome. It demonstrated no relative benefits of linagliptin vs glimepiride, when given in addition to the usual standard of care, for the primary composite outcome of cardiovascular death, non-fatal myocardial infarction and non-fatal stroke (HR 0.98 [95% CI 0.84, 1.13]), and fatal/non-fatal stroke (0.86 [0.66, 1.12]) [27]. CAROLINA also implemented a cognition substudy to assess relative effects on global, and domain-specific, cognitive function [28], and herein we report the impact on cognitive decline and on global cognitive function using a validated standardised cognitive test battery sensitive to relatively mild cognitive changes.

## Methods

### Trial design and participants

The CAROLINA-COGNITION study was an integral part of the CAROLINA trial, a randomised, active comparator, double-blind study to evaluate the cardiovascular safety of linagliptin 5 mg vs glimepiride 1–4 mg, and was event driven until a minimum of 631 patients had experienced the primary outcome event. Over 600 centres across 43 countries participated, enrolling adults with relatively early type 2 diabetes (HbA_1c_ 48–69 mmol/mol [6.5–8.5%]) at elevated risk of cardiovascular events receiving usual care while excluding those already on insulin therapy [27]. Elevated cardiovascular risk was defined as: previous vascular disease and/or evidence of vascular-related end-organ damage and/or age ≥70 and/or a minimum of two predefined cardiovascular risk factors. Detailed inclusion and exclusion criteria have previously been published [27] and are available in the supplementary material (ESM Table 2).

Glimepiride was initiated at 1 mg and up-titrated every 4 weeks during the first 16 weeks to a maximal dose of 4 mg. Investigators were encouraged to use glucose-lowering rescue medication if glycaemic control was insufficient and to manage all other cardiovascular risk factors according to applicable guidelines and current standards of care.

### CAROLINA-COGNITION study

The cognition substudy aimed to investigate if treatment with linagliptin compared with glimepiride, once daily on top of standard care, could prevent ACD [28]. The study protocol was approved by the institutional review board or independent ethics committee from each site, and all participants provided written informed consent. Because of the cognitive test procedures, participants were required to live in a country that had a native language built on the Latin alphabet. Participants were included in the primary analysis if: (1) their years of formal education were captured; (2) they had a valid baseline cognitive assessment; and (3) they had a valid follow-up cognitive assessment within 7 days after their last study-drug intake. Those with Mini-Mental State Examination (MMSE) score <24 at baseline were excluded, since the cognition substudy intended to investigate if treatment with linagliptin could prevent occurrence of cognitive decline.

### Procedures

A comprehensive description of the cognitive assessment and corresponding procedures has previously been published [28]. In brief, to measure ACD, two cognitive measures were used: (1) the MMSE, a widely used and validated screening test for global cognition in older adults [29], for which a score below 24 is generally accepted to indicate cognitive impairment [30]; and (2) a more sensitive, domain-specific composite measure of attention and executive functioning combining performance on both Verbal Fluency Test (VFT) and Trail Making Test (TMT) [28, 31, 32]. For the VFT, F – A – S were used for letter fluency, and animals for category.

As depression is a known confounder of cognitive performance, participants were also asked to complete the Center for Epidemiologic Studies Depression Scale (CES-D), a 20-item questionnaire on depressive symptoms experienced over the last week, during each visit [33]. A score ≥16 indicates presence of depressive symptoms.

Further details around the MMSE, attention and executive functioning score and CES-D have been published previously [28]. Additional details on the attention and executive functioning derivation are also described in ESM Methods. All tests were administered by trained investigators, or designated site personnel.

### Time-windows

Cognitive assessments were conducted at baseline, after 160 weeks of follow-up and at end of follow-up provided that an MMSE score ≥24 was obtained at baseline. End of follow-up occurred either when a participant discontinued study medication/discontinued trial or at the end of the main trial (see ESM Fig. 1 for description of handling of missing cognitive assessments).

### Cognitive outcomes

#### Primary cognitive outcome

The primary cognitive outcome was defined as the incidence of ACD at end of follow-up, using a regression-based index (RBI). The RBI score reflects the difference between the observed and predicted cognitive score for each individual, and takes potential confounders (i.e. baseline test performance, age, years of formal education, sex, race and test-retest interval) into account at subject level, as opposed to raw change in test scores. The derivation and calculation of the RBI is previously described [28] and additional information on the calculation of the predicted score can be found in the supplementary material (ESM Methods). Participants were classified as having ACD when their cognitive decline score was at or below the 16th percentile of the RBI score of the total substudy population: this cut-off was chosen as it corresponds approximately to one SD below the mean. Participants with a valid baseline cognitive assessment who did not understand the cognitive test instructions at follow-up were also classified as having ACD. The ACD classification thus identifies individuals that decline faster than would be expected compared with other participants, while considering the confounders listed above at an individual level. Considerations for handling potential protocol deviations and missing data points are described in the supplementary material (ESM Table 3). A secondary analysis, to check the robustness of the results, was also performed, using the 10th percentile as cut-off (instead of the 16th percentile) to define ACD. Predefined sensitivity analyses, post hoc analyses, handling of potential protocol deviations and missing data are described in ESM Table 3, ESM Fig. 1 and ESM Fig. 2.

#### Subgroup analyses

Subgroup analyses similar to the primary analysis of the primary cognitive outcome were predefined for the following baseline variables: sex (male/female), age (<70, ≥70 years), race (black, white, Asian, other), ethnicity (Latino/Hispanic, non-Latino/Hispanic), CES-D score (<16, ≥16 and median split), duration of type 2 diabetes (≤1 year, >1 to ≤5 years, >5 to ≤10 years, >10 years), urine albumin/creatinine ratio (UACR) (<30 mg/mmol, ≥30 mg/mmol to ≤300 mg/mmol, >300 mg/mmol), and cardiovascular risk categories (high, moderate–high, moderate–low or low).

A post hoc subgroup analysis for the primary cognitive outcome was also conducted for participants with age <75 vs ≥75 years.

#### Secondary cognitive endpoints

Secondary cognitive endpoints include: (1) the incidence of ACD at end of follow-up, using the 10th percentile; (2) the incidence of ACD after 160 weeks of follow-up, both with the 16th and the 10th percentile; and (3) the incidence of ACD using the 16th percentile of the z score of the cognitive measures (i.e. MMSE and attention and executive function) instead of RBI score, both at end of follow-up and after 160 weeks.

#### Further cognitive endpoints

Further predefined endpoints included: (1) the incidence of ACD defined as an MMSE score <24 or a decline of >4 points on MMSE, relative to baseline; (2) incidence of depression defined as a CES-D score of ≥16; and (3) changes from baseline for all cognitive (sub)tests of VFT, TMT and MMSE. All endpoints were derived after 160 weeks of follow-up and at end of follow-up for both treatment groups.

### Post hoc assessment of metabolic response and hypoglycaemia

Change from baseline for HbA_1c_ and weight was analysed, as was the occurrence of hypoglycaemia. The latter was not adjudicated, but reported by investigators based on participant symptoms or laboratory values according to: (1) symptomatic hypoglycaemic adverse event (AE) with plasma glucose ≤3.9 mmol/l, or hypoglycaemic AE with plasma glucose <3.0 mmol/l; (2) severe hypoglycaemic AE defined as requiring the assistance of another person to actively administer carbohydrate, glucagon or other resuscitative actions; or (3) hypoglycaemic AE leading to hospitalisation.

### Statistical analyses

For the analysis of the primary and secondary endpoints, the incidence of ACD at end of follow-up was compared between treatment groups using a logistic regression model including treatment as a factor. The OR, along with the 95% Wald CI and two-sided *p* value, were calculated for treatment comparison. For subgroup analyses, terms for treatment, subgroup, and subgroup-by-treatment interactions were included in the logistic regression model.

The continuous cognition endpoints, change from baseline, were analysed by a linear mixed-effects model for repeated measures. The restricted maximum likelihood estimation method was involved, and the Kenward–Roger method was used to adjust standard errors and estimate denominator *df*. For the pairwise comparisons of linagliptin vs glimepiride, the differences between the expected means were estimated by the difference in the corresponding adjusted means. Two-sided 95% CIs based on the t distribution were also computed. Data after 160 weeks of follow-up and at end of follow-up were included in these analyses. In all analyses, participants were grouped by original random treatment assignment.

The repeated-measures analysis described above was also applied to change from baseline over time in HbA_1c_ and weight. Data were included up to the planned week that could theoretically be achieved by all participants.

Hypoglycaemic events that occurred during treatment, or within 7 days after the last dose of a study drug, were analysed as time-to-first-event using a Cox proportional hazards model, with treatment as a factor (two-sided *p* value from Wald’s χ^2^ test).

All analyses were conducted with SAS version 9.4 (SAS Institute, Cary, NC, USA).

### Sample size considerations

For the primary outcome it was expected that approximately 20–22% (ESM Methods) of the whole cohort would meet the criteria for ACD [23]. Assuming at least 4500 participants at baseline, with cognitive follow-up, this would allow the detection of an RR reduction of 20% in the linagliptin group relative to the glimepiride group with a power of 80% and two-sided α of 0.05.

## Results

Out of 4529 participants from countries eligible for the CAROLINA-COGNITION study, 4018 were included at baseline. Reasons for exclusion at baseline were missing information on education level or language or illiteracy (*n* = 232), missing MMSE or MMSE <24 (*n* = 279). In total 3163 out of 4018 participants from 31 countries (ESM Fig. 2 and ESM Fig. 3) had at least one valid follow-up cognitive assessment and were included in the primary analysis. Reasons for follow-up assessment being missing, or excluded (following predefined criteria) [23], were: no valid follow-up assessment of cognition at any visit (*n* = 566, of whom *n* = 161 died before their scheduled assessment); and the only available cognitive assessment being more than 7 days after their last medication intake (*n* = 289).

Baseline clinical characteristics and cognitive scores were well balanced between the two treatment arms (Table 1). Overall, mean±SD age was 64.4 ± 9.2 years (14.2% ≥ 75 years), mean duration of type 2 diabetes 7.6 ± 6.1 years, and mean years of formal education 10.8±3.5. Mean BMI was 30.8 ± 5.0 kg/m^2^ and HbA_1c_ 54 ± 6.1 mmol/mol (7.1 ± 0.6%). Mean MMSE at baseline was 28.5 ± 1.7 and CES-D 9.0 ± 8.2 with 16.7% scoring CES-D ≥ 16.

**Table 1:** Baseline characteristics by treatment group

Variables	Linagliptin (*n* = 1618)	Glimepiride (*n* = 1545)
Male/female	1002 (61.9)/616 (38.1)	958 (62.0)/587 (38.0)
Age, years	64.4 ± 9.1	64.4 ± 9.3
Medical history		
History of myocardial infarction	226 (14.0)	187 (12.1)
History of cerebrovascular disease	169 (10.4)	154 (10.0)
Atrial fibrillation	86 (5.3)	74 (4.8)
Known coronary artery disease	394 (24.4)	366 (23.7)
Education level, years	10.8±3.4	10.8±3.5
BMI, kg/m^2^	30.8±5.0	30.7±4.9
MMSE score	28.5±1.7	28.5±1.7
Depression score according to CES-D	8.7±8.0	9.3±8.3
< 16	1335 (82.5)	1242 (80.4)
≥ 16	250 (15.5)	278 (18.0)
Missing	33 (2.0)	25 (1.6)
eGFR (MDRD), ml min^−1^ [1.73 m]^−2^	75.8±19.0	76.9±18.8
Type 2 diabetes duration, years	7.7±6.2	7.4±5.9
HbA_1c_, mmol/mol (%)	54.3±6.0 (7.1±0.5)	54.5±6.2 (7.1±0.6)
Fasting plasma glucose, mmol/l	7.8±1.7	7.8±1.6
Glucose-lowering medications		
Metformin	1348 (83.3)	1306 (84.5)
Sulfonylurea	434 (26.8)	422 (27.3)
Glinide	13 (0.8)	13 (0.8)
α-glucosidase inhibitor	43 (2.7)	34 (2.2)
Thiazolidinedione	1 (0.1)	2 (0.1)
Cardiovascular medications		
Lipid-lowering	1197 (74.0)	1180 (76.4)
Statins	1111 (68.7)	1113 (72.0)
Antihypertensives	1428 (88.3)	1387 (89.8)
Systolic BP, mmHg	135.9±15.9	136.2±16.4
Diastolic BP, mmHg	78.8±9.5	78.8±9.3
LDL-cholesterol, mmol/l	2.4±0.9	2.4±0.9

Data are *n* (%) or mean±SD unless otherwise stated

MDRD, Modification of Diet in Renal Disease study equation

Median (min–max) time between randomisation and assessment at end of follow-up was 6.12 (0.02–7.42) years, with no significant differences between treatment arms. Baseline characteristics of participants who dropped out of the substudy without having a post-baseline assessment were balanced between treatment groups (ESM Table 4) and their profile differed only modestly from the overall population with slightly more prevalent cardiovascular disease at baseline and slightly higher proportion reporting depressive symptoms.

### Primary cognitive outcome

**Fig. 1:**
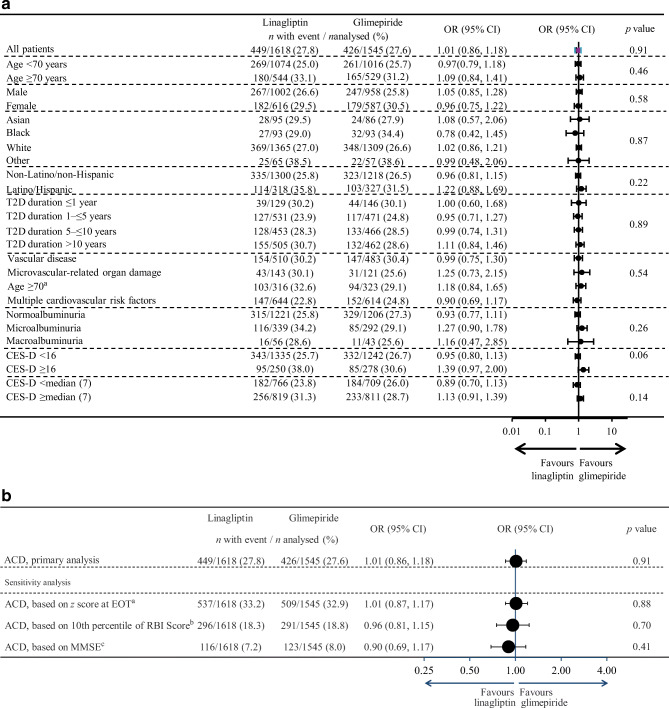
Effect on ACD with linagliptin vs glimepiride at end of treatment. (**a**) Primary analysis and effects in prespecified subgroups. *p* values depict overall treatment effect (top value) or treatment-by-subgroup interaction. ^a^The lower age-group relates to number of participants enrolled specifically according to the age-specific inclusion-criterion. (**b**) Secondary analysis of effects on ACD with linagliptin vs glimepiride. Treatment group values are *n* with event/*n* analysed (%). EOT, end of treatment, T2D, type 2 diabetes. ^a^Incidence of ACD based on 16th percentile of z score for MMSE and/or attention and executive functioning. ^b^Incidence of ACD based on 10th percentile of RBI score. ^c^Incidence of ACD at EOT based on MMSE score of <24 or a decline of >4 points in MMSE score at EOT relative to baseline

Of the 3163 participants that were included in the primary analysis, 449/1618 (27.8%) in the linagliptin group and 426/1545 (27.6%) in the glimepiride group had ACD during follow-up. In those with ACD, the MMSE changed by −2.5 ± 3.6 points and the attention and executive function z score by −0.7 ± 1.0 points, as opposed to 0.4 ± 1.6 and 0.1 ± 0.7 in those without ACD (ESM Table 5). There was no difference in occurrence of ACD between treatment arms (1.01 [95% CI 0.86, 1.18]), nor in the subgroup analyses (Fig. 1a), although in both groups, the proportion with ACD was higher in those with higher age, in women, in those with Latino/Hispanic ethnicity, in those with vascular disease, and in those with higher CES-D scores at baseline. In a post hoc defined analysis amongst those ≥75 years of age, ACD occurred in 82/226 (36.3%) in the linagliptin group and 67/224 (29.9%) in the glimepiride group (1.33 [0.90, 1.98]).

Sensitivity analyses supported the results of the primary analysis (Fig. 1b, ESM Table 6).

### Secondary and further cognitive outcomes

The analysis of ACD after 160 weeks of follow-up showed consistent results with the primary analysis (linagliptin: 446/1618 [27.6%], glimepiride: 406/1545 [26.3%], 1.07 [95% CI 0.91, 1.25]). Similar results were found with the secondary analysis applying the 10th percentile as cut-off for the RBI score at end of follow-up (linagliptin 296/1618 [18.3%], glimepiride 291/1545 [18.8%], 0.96 [0.81, 1.15]), (ESM Tables 6–8). Also, no differences were found between the treatment arms in the subgroups (Fig. 1a), or in a sensitivity analysis including 3452 participants (linagliptin: 1766, glimepiride: 1686), regardless of whether their latest post-baseline assessment was conducted later than 7 days after treatment discontinuation (1.04 [0.89, 1.21]) (ESM Fig. 2).

Absolute changes from baseline in MMSE, VFT and TMT scores were similar between treatment groups, both after 160 weeks and at end of follow-up (Table 2).

**Table 2:** Absolute changes in MMSE, attention and executive function, TMT, VFT and CES-D by treatment

	Week 160^a^		End of follow-up^b^	
	Linagliptin (*n* = 1618)	Glimepiride (*n* = 1545)	Linagliptin (*n* = 1618)	Glimepiride (*n* = 1545)
MMSE score				
Baseline	28.5±1.7	28.5±1.7	28.5±1.7	28.5±1.7
Follow-up	28.2±2.1	28.3±2.2	28.1±2.7	28.1±2.7
Change from baseline	−0.2±2.0	−0.2±2.1	−0.4±2.6	−0.4±2.6
A&E (*z* score)^c^				
Baseline	−0.01±0.70	0.01±0.01	−0.01±0.70	0.01±0.01
Follow-up	−0.06±0.73	−0.05±0.71	−0.10±0.77	−0.09±0.78
Change from baseline	−0.05±0.74	−0.06±0.72	−0.11±0.81	−0.14±0.81
VFT letter 60 (in seconds)^d^				
Baseline	9.2±4.2	9.3±4.1	9.2±4.2	9.3±4.1
Follow-up	8.9±4.0	9.1±4.0	9.1±4.3	9.1±4.5
Change from baseline	−0.2±2.6	−0.2±2.7	−0.2±2.9	−0.2±3.4
VFT animals 60 (in seconds)^e^				
Baseline	16.1±6.6	16.0±6.2	16.1±6.6	16.0±6.2
Follow-up	15.6±6.6	15.6±6.5	15.7±7.5	15.4±7.1
Change from baseline	−0.5±6.0	−0.4±5.7	−0.6±7.4	−0.6±6.7
Overall VFT 60 (*z* score)				
Baseline	−0.01±0.89	0.002±0.85	−0.01±0.89	0.002±0.85
Follow-up	−0.09±0.84	−0.07±0.84	−0.06±0.96	−0.08±0.96
Change from baseline	−0.07±0.63	−0.06±0.64	−0.07±0.78	−0.09±0.76
TMT-A (in seconds)				
Baseline	51.2±28.1	50.4±26.7	51.2±28.1	50.4±26.7
Follow-up	53.7±30.3	53.3±30.6	54.1±30.6	55.4±33.3
Change from baseline	2.1±24.2	2.8±25.4	3.4±25.6	4.4±28.1
TMT-B (in seconds)				
Baseline	113.1±57.8	111.8±61.3	113.1±57.8	111.8±61.3
Follow-up	118.2±64.4	118.3±63.8	125.5±67.7	125.3±66.0
Change from baseline	7.1±53.9	9.8±54.1	15.2±63.3	18.2±59.5
TMT ratio^f^				
Baseline	1.4±1.0	1.4±1.0	1.4±1.0	1.4±1.0
Follow-up	1.4±1.0	1.4±1.0	1.6±1.1	1.5±1.0
Change from baseline	0.1±1.1	0.2±1.1	0.2±1.3	0.3±1.0
CES-D score				
Baseline	8.7±8.0	9.3±8.3	8.7±8.0	9.3±8.3
Follow-up	10.5±9.9	11.4±10.6	10.2±9.9	10.6±9.6
Change from baseline	1.7±9.6	2.1±10.4	1.6±9.6	1.4±10.0

Data are mean±SD

Time between randomisation and visit: median (min–max): ^a^3.07 (0.02–3.19), ^b^6.40 (3.20–7.42) years

^c^A&E, composite *z* score for attention and executive functioning

^d^VFT letter 60: averaged VFT scores for the letters F, A and S in 60 s

^e^VFT animals 60: VFT score for the category animals in 60 s

^f^TMT ratio: (TMT-B − TMT-A)/TMT-A

The proportion of participants experiencing substantial depressive symptoms (CES-D ≥ 16) after 160 weeks of follow-up was lower for those who received linagliptin (22.9%) compared with glimepiride (26.1%), 0.84 (0.71, 1.00). However, this difference was attenuated at the end of follow-up (linagliptin: 21.3%, glimepiride: 23.8%, 0.86 [0.71, 1.05]).

### Effects on metabolic variables and hypoglycaemia

The mean (±SD) dose of glimepiride over the trial duration was 2.9 ± 1.1 mg daily. Consistent with the overall population [27], HbA_1c_ over time did not differ between treatment groups in the CAROLINA-COGNITION study (ESM Fig. 4), although some initial differences were noted, e.g., adjusted mean (95% CI) difference between linagliptin and glimepiride at week 16 and week 256: 2.84 (2.37, 3.32) mmol/mol (0.26 [0.22, 0.30]%), and 0.02 (−0.96, 1.00) mmol/mol (0.00 [−0.09, 0.09]%), respectively. Modest weight gain was observed in the glimepiride group early in the study and maintained thereafter, e.g., adjusted mean (95% CI) difference between linagliptin and glimepiride at week 16 and week 256: −1.42 (−1.61, −1.22) kg, and −1.77 (−2.22, −1.33) kg, respectively (ESM Fig. 5).

The incidence of hypoglycaemic events was lower with linagliptin across all hypoglycaemia categories in a post hoc analysis (ESM Fig. 6). Rates of symptomatic investigator-defined hypoglycaemia events with plasma glucose ≤3.9 mmol/l, plasma glucose <3.1 mmol/l, or severe hypoglycaemic events were 1.30 events per 100 participant years for linagliptin and 8.61 per 100 participant years for glimepiride (HR 0.17 [95% CI 0.14, 0.20]); rates of severe hypoglycaemic events were 0.07 and 0.48 per 100 participant years, respectively (0.14 [0.06, 0.32]). There were very few hospitalisations due to hypoglycaemic events (one event in the linagliptin group and 15 events in the glimepiride group; 0.01 events per 100 participant years for linagliptin and 0.17 per 100 participant years for glimepiride; 0.06 [<0.01, 0.48]).

## Discussion

In this comparative cognitive interventional trial of 3163 individuals with relatively early type 2 diabetes without impaired cognition at baseline, and elevated cardiovascular risk, the effects of linagliptin 5 mg or glimepiride 1–4 mg did not differ for the risk of ACD when assessed using three different instruments over a median follow-up of 6.12 years. This result was observed in the setting of glycaemic equipoise between treatment groups, but with differing effects on hypoglycaemia and weight.

These findings have biological implications as, in the context of neutral effects on ACD with linagliptin compared with placebo [26], they do not support previous hypotheses that DPP-4 inhibitors or sulfonylureas may affect cognitive function positively. However, the neutral effect on cognitive function also provides reassurance that these medications do not differentially affect cognitive performance, which has been debated for other glucose-lowering medications, e.g., metformin and insulin, albeit with conflicting data [14–17].

The duration of the substudy was driven by the number of cardiovascular events in the main study, and this led to a reasonably long observation period. As the progress of cognitive decline is usually slow, this is a strength of the present substudy. Another strength is the consistency using the more stringent 10th percentile as cut-off for ACD. With this cut-off we captured a group with substantial cognitive decline (i.e. >3 MMSE points on average). The data also show that in the group without ACD, according to our definitions, cognitive test performance was essentially stable over the course of 6 years, highlighting the importance of a dichotomous rather than continuous cognitive outcome measure for a trial if one intends to prevent clinically meaningful cognitive decline. Clearly a clinical diagnosis of dementia or MCI, based on a full diagnostic evaluation combined with elaborate cognitive testing and ideally adjudication, would have been even more meaningful as an outcome measure, however this is currently not feasible in a large international outcome trial such as CAROLINA, and therefore is a limitation of this substudy. Moreover, the CAROLINA-COGNITION study involved the MMSE, TMT and VFT as the cognitive testing battery, and it is uncertain if results would have looked different using other testing batteries or subjective cognitive complaints measures [24, 34].

The number of participants analysed both with global cognitive functioning and domain-specific function items was relatively large, and although the ADVANCE trial had a larger number of participants (*n*=11,132), this was not a blinded placebo-controlled trial, it was designed to assess effects of intensive glucose lowering on cognitive impairment, and only used MMSE [8]. Prior to CAROLINA-COGNITION, there had been very few RCTs that have been designed to investigate non-glycaemic effects of pharmacological interventions on cognition in type 2 diabetes in a direct comparative way, with most being too small or with too short duration to adequately address this question [13, 25]. In CAROLINA, no difference in HbA_1c_ was observed. However, there was a marked difference in risk for hypoglycaemia across treatment arms, which is of relevance since hypoglycaemia, in particular severe events, has been correlated with cognitive impairment [23]. The role of hypoglycaemia in relation to the risk of MCI and dementia is, however, debated [24]. In the present substudy, there were very few episodes of severe hypoglycaemia, and too few to reliably analyse this relationship, and whether the results would have looked different in populations more susceptible to hypoglycaemia is unknown. Also, while greater weight in mid-life is associated with poorer cognitive function in individuals with type 2 diabetes [35], the cognitive function tests applied might not be sufficiently sensitive to the relatively modest magnitude of weight changes observed in this trial.

It should be noted that we observed a relatively large difference between numbers of participants with baseline assessment (*n* = 4529) and follow-up assessment (*n* = 3163). The main reason for this was due to protocol specification not to include cognitive assessments more than 7 days after end of treatment. In addition, several participants prematurely discontinued the study drug, at a rate also observed in other trials, and some died, which was part of the primary outcome of the trial. Nonetheless, rates were equally distributed across both treatment arms and the neutral result was reflected in subgroups and in sensitivity analyses, including using the broader participant dataset (*n* = 3452; 86% of those included at baseline). However, the generalisability of these results does not necessarily extend to those at higher risk for cognitive decline, or to those with MMSE <24, and we also cannot extrapolate the results to individuals with more advanced type 2 diabetes, or to other DPP-4 inhibitors or sulfonylureas.

An interesting exploratory observation from this substudy was that approximately a quarter of the population had shown signs of depressive symptoms at week 160, and fewer in the linagliptin group (OR 0.84 [0.71, 1.00]), a finding that was attenuated at end of follow-up (OR 0.86 [0.71, 1.05]). Although both differing hypoglycaemia burden and weight gain could play a role [36], this substudy cannot provide definitive answers, and further studies are required.

In conclusion, in a population with relatively early type 2 diabetes, with intact cognitive function at baseline and at elevated risk for cardiovascular events, there was no difference in the rate of ACD between those treated with linagliptin or glimepiride. Additionally, this trial demonstrated that cognitive assessment in a large international outcome trial is feasible.

## Supplementary Information

ESM (PDF 1777 kb)

## Data Availability

The datasets generated during and/or analysed during the current study are available from the corresponding author on reasonable request.

## Abbreviations

ACD: Accelerated cognitive decline
AE: Adverse event
CES-D: Center for Epidemiologic Studies Depression Scale
DPP-4: Dipeptidyl peptidase-4
GLP-1: Glucagon-like peptide-1
MCI: Mild cognitive impairment
MMSE: Mini-Mental State Examination
RBI: Regression-based index
TMT: Trail making test
UACR: Urine albumin/creatinine ratio
VFT: Verbal fluency test
