# Supplementary material for: Effects of linagliptin vs glimepiride on cognitive performance in type 2 diabetes: results of the randomised double-blind, active-controlled CAROLINA-COGNITION study
Source: Diabetologia. 2021 Feb 9;64(6):1235–45. doi: 10.1007/s00125-021-05393-8 (PMC8099814; doi:10.1007/s00125-021-05393-8)
Supplement: Supplementary file 1 — (PDF 1777 kb) [file 125_2021_5393_MOESM1_ESM.pdf]

Electronic supplementary material (ESM)

Biessels GJ, Verhagen C, Janssen J, van den Berg E, Wallenstein G, Zinman B, Espeland MA, Johansen OE.
Effects of linagliptin versus glimepiride on cognitive performance in type 2 diabetes: results of the randomised double-blind, active-controlled CAROLINA-COGNITION study

Section		Page
ESM Methods	Description of derivation of A&E scores and regression methods used to derive predicted scores and power considerations	2
ESM Table 1	Randomised trials involving DPP-4 inhibitors or sulphonylureas designed to test cognitive outcomes in type 2 diabetes	3
ESM Table 2	Key inclusion criteria CAROLINA®	5
ESM Table 3	Handling of protocol deviations and missing data points	7
ESM Table 4	Baseline characteristics for patients dropping out of the study post-baseline.	8
ESM Table 5	Changes in MMSE, A&E z-score, TMT and VFT by accelerated cognitive decline (yes/no) regardless of treatment group at week 160 and end of follow-up.	10
ESM Table 6	Results of the primary and secondary analyses for accelerated cognitive decline (ACD)	12
ESM Table 7	Changes in cognitive or depression scores after 160 weeks of follow-up	13
ESM Table 8	Changes in cognitive or depression scores at end of follow-up	14
ESM Figure 1	Handling of missing cognitive assessments	15
ESM Figure 2	Flowchart for CAROLINA-COGNITION study	16
ESM Figure 3	Participating countries in CAROLINA-COGNITION study	17
ESM Figure 4.	HbA1c over time in participants in the CAROLINA-COGNITION study	18
ESM Figure 5	Weight over time in participants in the CAROLINA-COGNITION study	19
ESM Figure 6	Proportion of participants in the CAROLINA-COGNITION study with hypoglycaemia	20

ESM Methods

Description of A&E derivation and regression methods used to derive predicted scores

A&E derivation

1) The VFT scores for the letters F, A and S in 60 s were averaged to one VFT letter fluency score.

2) Because of word-frequency differences between different Latin-based languages, correction factors per language were calculated based on all baseline VFT scores of patients included in baseline primary analysis population (③ in ESM Figure 2).

3) The effect of language on VFT scores was evaluated by applying an ANCOVA model with fixed effects for language and covariates for age, gender, race and years of formal education to each of the 4 VFT scores (FAS in 15 seconds and in 60 seconds, and the category animals in 15 seconds and in 60 seconds). Correction factors were calculated by dividing the LS Mean of the specific language by the LS Mean of the reference language (LSmean language/ LSmean English). Each individual VFT score was corrected for the influence of language by multiplying the individual observed score by the calculated correction factor for the language in which the test was performed, so that all VFT scores for a category are comparable. The same correction factors were applied to baseline as well as to scores obtained post-baseline.

4) The TMT ratio was calculated, providing an index for executive functioning: (TMT B –TMT A) / TMT A.

5) After correction, the scores were converted into z-scores. Z-scores are used to standardize raw test scores and make them directly comparable. Z-scores were calculated as follows: individuals z-score = individuals test score – mean score of sample)/ standard deviation of sample. TMT and VFT scores were converted into z-scores. The z-scores of the category fluency (animals) and the letter fluency (F, A and S) were averaged to form an overall VFT z-score.

Because the TMT and the VFT both tap the domain "attention and executive functioning" (A&E) the z-scores on these two tests were averaged: A&E z-score = mean (overall VFT z-score 60 seconds, -TMT ratio z-score). The negated TMT ratio and VFT overall z-score was averaged to compose one score for attention and executive functioning. The TMT ratio z-score was inverted, since higher scores mean lower performance. In secondary analysis the TMT and VFT were analyzed separately to control for potential test-specific effects.

Regression methods used to develop predicted scores

Predicted follow-up scores were calculated for each individual by means of an ANCOVA model. The following covariates (predictors) were added to the model: baseline performance, age, education, gender, race and test-retest interval. To derive the individual predicted follow-up scores, regression coefficients obtained from the ANCOVA model is multiplied by individual values on each predictor using the equation: FU predict = $a + \beta 1 \times P1 + \beta 2 \times P2 + \ldots + \beta n \times Pn$, where a is the intercept and $\beta 1$ is the regression coefficient for predictor P1, $\beta 2$ for predictor P2 and βn for predictor Pn.

Power considerations

Accelerated cognitive decline was defined as an RBI score within the lowest 16% for the MMSE and/or A&E RBI score. This set was composed of three subsets of patients:

i. Patients with ACD in MMSE and the A&E RBI, assumed to account for 2/3 of patients with ACD
ii. Patients with ACD only in MMSE, assumed to account for 1/3 of patients with ACD
iii. Patients with ACD only in A&E RBI, assumed to account for 1/3 of patients with ACD

We assumed that 2/3 of the patients with ACD on either the MMSE or A&E would also have an impairment on the other test (subset i), which would constitute 2/3*16% of the study population, leaving 1/3*16% each for people falling in subsets ii and iii 1/3. The union of these subsets thus consists of (2/3+1/3+1/3)*16% that is equal to 21.3%, which we therefore suggested to represent a range of 20 to 22% of the expected occurrence of the primary outcome event (Biessels GJ, et al. BMC Neurology 2018; 18: 7).

ESM Table 1. List of randomised trials involving DPP-4 inhibitors or sulphonylureas designed to test cognitive outcomes in type 2 diabetes.

**Trial**	**Inclusion criteria**	***n***	**Intervention**	Outcome measure	**Result[1]**	**Risk of bias**	**Quality of evidence[2]**
Abbatecola 2006 [1]	Treatment-naïve participants aged 60-78 years, from university outpatient offices.	156	Glibenclamide (glyburide)<br>vs<br>repaglinide	Global cognitive function with MMSE at 12 months<br>Composite score of attention and executive functioning (TMT, VFT and digit span) at 12 months	MMSE difference 0.90 (95% CI: 0.12, 1.68) (i.e. small effect in favour of glibenclamide)<br>MD: 0.00 (95% CI: −0.01, 0.01)	Unclear (no description of random sequence generation, allocation concealment, blinding of participants and personnel and selective reporting)	Low (risk of bias and single study with small sample size)
Ryan 2006 [2]	Adults receiving metformin combination therapy and HbA1c ≤ 64 mmol/mol (8%), BMI ≤ 27 kg/m^2, no evidence of dementia (MMSE ≥ 27) no evidence of depression according to mini international neuropsychiatric interview (MINI).	145	Rosiglitazone + metformin<br>vs<br>glibenclamide (glyburide) + metformin	Cognitive test scores (DSST, RAVLT immediate, CANTAB) after 24 weeks<br><br>Composite scores for the cognitive domains: working memory, learning ability and cognitive efficiency after 24 weeks	DSST: MD: −0.20 (95% CI −0.50, -0.10)<br>RAVLT immediate: MD −1.40 (95% CI −1.70, −1.10) in favor of rosiglitazone<br>CANTAB:<br>1) small numerical advantages for rosiglitazone plus metformin<br>on the items of Paired Associates Learning, Pattern Recognition Memory and Rapid Visual Information Processing.<br>2) small numerical advantage<br>for glibenclamide (glyburide) plus metformin<br>on the item Reaction Time<br>No differences on composite scores	Unclear (no methods for allocation, blinding of participants and personnel identified, no protocol identified)	Low (risk of bias, single study with small sample size, imprecision)
ADVANCE 2008 [3]	≥ 55 years, ≥ 30 years, history of major macro- or micro-vascular disease or at least one other risk factor for vascular disease	11140	Intensive glucose control (gliclazide), discontinued other sulphonylureas, plus other treatments, to achieve HbA1c of ≤ 48 mmol/mol (6.5%)<br>vs<br>standard-control (former gliclazide use, required to substitute with other sulphonylurea with target HbA1c levels from local guidelines)	Number of participants with cognitive decline on MMSE (3 or more points) (yes/no)<br><br>Incidence of dementia (based on DSM-IV criteria)<br>After median follow-up duration of 5 years.	RR 0.98 (0.88, 1.08) (i.e. no difference between groups)<br><br>RR 1.27 (0.87, 1.85) (i.e. no differences between groups)	Unclear (no description in random sequence generation, allocation concealment and blinding of participants and personnel)	Moderate (risk of bias)<br><br>Low (risk of bias and low event rate; 61 intervention vs 48 control)

Perna 2018 [4]	Age > 65 years<br><br>Healthy cognition (exclusion: MMSE ≥ 27)<br><br>Absence of neurological disease, mental disorders, sensorial impairment and alcoholism	39	SGLT2-inhibitor (on top of max. dose metformin as stable regime)<br><br>vs<br><br>Incretins (on top of max. dose metformin as stable regime)	Differences between groups on mean cognitive change on VFT, AMT BSRT.<br><br>After 52 weeks	VFT: –0.65 (–1.86, -0.56)<br><br>AMT: –0.34 (–1.64, 0.96)<br><br>BSRT: –3.57 (–8.80, 1.66)	High risk because of lack of allocation concealment. Unclear risk because no methodology for blinding of participants, personnel and outcome assessors.	Low (risk of bias, single study with small sample size, imprecision for some cognitive outcomes)
Biessels 2019 [5]	High cardiovascular and renal risk	1545	Linagliptin<br><br>vs<br><br>placebo<br><br>(on top of standard care)	Incidence of accelerated cognitive decline with RBI ≤ 16th percentile on either MMSE or attention and executive composite z-score (VFT and/or TMT ratio) after median follow-up duration of 2.5 years	OR 0.96 (95% CI: 0.77, 1.19)	Low (note: substantial drop-out of > 30%, however, inherent to study design (high CV risk), drop-out was balanced between treatment arms, reasons were reported, and neutral outcome)	Moderate (relative short follow-up duration)

[1] Results for intervention group. [2] Evaluation according to GRADE (Working Group grades of evidence): High quality: further research is very unlikely to change our confidence in the estimate of effect. Moderate quality: further research is likely to have an important impact on our confidence in the estimate of effect and may change the estimate. Low quality: further research is very likely to have an important impact on our confidence in the estimate of effect and is likely to change the estimate. Very low quality: we are very uncertain about the estimate. Where applicable, evaluation of risk of bias and quality of evidence is adapted from Areosa Sastre et al. [6]. MD: mean difference, RR: relative risk, OR: odds ratio, CANTAB: Cambridge Neuropsychological Test Automated Battery. DSST: Digit Symbol Substitution Test RAVLT: Rey Auditory Verbal Learning Test, MMSE: Mini-Mental State Examination, DSM-IV: Diagnostic and Statistical Manual of Mental Disorders. RBI: regression based index score. VFT: verbal fluency test. TMT: trail making test. AMT: Attentive Matrices Test. BSRT: Babcock Story Recall Test.

References.

1. Abbatecola AM, Rizzo MR, Barbieri M, et al. Postprandial plasma glucose excursions and cognitive functioning in aged type 2 diabetics. Neurology 2006;67:235-40
2. Ryan CM, Freed MI, Rood JA, Cobitz AR, Waterhouse BR, Strachan MW. Improving metabolic control leads to better working memory in adults with type 2 diabetes. Diabetes Care 2006;29:345-51
3. de Galan BE, Zoungas S, Chalmers J, et al. Cognitive function and risks of cardiovascular disease and hypoglycaemia in patients with type 2 diabetes: the Action in Diabetes and Vascular Disease: Preterax and Diamicron Modified Release Controlled Evaluation (ADVANCE) trial. Diabetologia 2009; 52: 2328–36
4. Perna S, Mainardi M, Astrone P, et al. 12-month effects of incretins versus SGLT2-Inhibitors on cognitive performance and metabolic profile. A randomized clinical trial in the elderly with Type-2 diabetes mellitus. Clin Pharmacol 2018;10:141-51
5. Biessels GJ, Verhagen C, Janssen J, et al. Effect of linagliptin on cognitive performance in patients with type 2 diabetes and cardiorenal comorbidities: the CARMELINA randomized trial. Diabetes Care 2019;42:1930-8
6. Areosa Sastre A, Vernooij RW, González-Colaço Harmand M, Martínez G. Effect of the treatment of Type 2 diabetes mellitus on the development of cognitive impairment and dementia. Cochrane Database Syst Rev. 2017 Jun 15;6:CD003804.

ESM Table 2. Key inclusion criteria CAROLINA®

Insufficient glycaemic control defined as one of the criteria (A or B) *AND*	Elevated risk of cardiovascular events defined as any (one or more) of the criteria (A, B, C or D)
(A) HbA1c 48–69 mmol/mol (6.5 - 8.5%) while patient is treatment naïve or treated with:	(A) Previous vascular disease:
(I) Metformin monotherapy	(I) MI (>6 weeks prior to informed consent IC)
(II) α-Glucosidase inhibitor monotherapy (e.g. acarbose, voglibose)	(II) Documented coronary artery disease (⩾50% luminal diameter narrowing of left main coronary artery or in at least two major coronary arteries in angiogram)
(III) Metformin plus α-glucosidase inhibitor (e.g. acarbose, voglibose)	(III) Percutaneous coronary intervention (>6 weeks prior to IC)
	(IV) Coronary artery bypass grafting (>4 years prior to IC) or with recurrent angina following surgery
	(V) Ischaemic or haemorrhagic stroke (>3 months prior to IC)
	(VI) Peripheral occlusive arterial disease
(B) HbA1c 48–58 mmol/mol (6.5 - 7.5%) while patient is treated with:	(B) Evidence of vascular-related end-organ damage:
(I) SU monotherapy	(I) Moderately impaired renal function (as defined by MDRD formula) with eGFR 30–59 ml/min/1.73 m^2
(II) Glinide monotherapy (e.g. repaglinide, nateglinide)	(II) Random spot urinary albumin:creatinine ratio ⩾30 μg/mg in two of three unrelated specimens in the previous 12 months.
(III) Metformin plus SU (for a maximum of 5 years)	(III) Proliferative retinopathy defined as retinal neovascularization or previous retinal laser coagulation therapy
(IV) Metformin plus glinide (for a maximum of 5 years)	
(V) α-Glucosidase inhibitor plus SU (for a maximum of 5 years)	C) Age ≥70 years
(VI) α-Glucosidase inhibitor plus glinide (for a maximum of 5 years)	D) At least two of the following cardiovascular risk factors:

	(I) Duration of type 2 diabetes >10 years<br>(II) Systolic BP >140 mmHg (or on at least 1 BP-lowering treatment) <6 months prior to IC<br>(III) Current daily cigarette smoking<br>(IV) LDL-cholesterol ⩾3.5 mmol/L (or specific current treatment for this lipid abnormality) <6 months prior to IC

CAROLINA: CARdiovascular Outcome Trial of LINAgliptin Versus Glimepiride in Type 2 Diabetes; IC: informed consent; BP: blood pressure; SU: sulphonylurea; MI: myocardial infarction; MDRD: modified diet in renal disease

ESM Table 3. Predefined sensitivity analyses

The table illustrates how potential or de facto protocol deviations and missing data points were handled through five sensitivity analyses.

		Sensitivity analysis
1.	Check the influence of inappropriate inclusion, potentially confounding co-morbid conditions and trial medication use (⑥ in ESM Figure 2)	Participants were excluded from the analysis if:<br>• major inclusion or exclusion criteria are violated<br>• incorrect trial medication is taken<br>• major neurological or psychiatric disease was present at baseline
2.	Check the influence of classifying participants who did not understand the instructions at follow-up as having accelerated cognitive decline	The last observation carried forward method was used for participants with missing MMSE and A&E RBI-scores at follow-up if the reason for missing is the inability of the participant to understand the instructions (instead of classifying them as having accelerated cognitive decline)
3.	Check for bias by differential lost to follow-up (worst-case scenario).	All participants with missing MMSE and A&E RBI-scores at follow-up were considered to have accelerated cognitive decline
4.	Check the impact of further baseline variables on the RBI score result, check for confounding by depression symptoms.	Age, gender, years of formal education, race, ethnicity and language and CES-D (score <16, ≥ 16) are included as covariates in the logistic regression analysis.
5.	Check for impact of the excluding participants that did not have their last post-baseline visit on treatment (i.e. ≤ 7 days after treatment stop, ④ in ESM Figure 2)	The latest post-baseline assessment was used as end of follow-up (regardless if participants were on-treatment). Time windows were not applicable here. Measurements before 700 days were not included.

ESM Table 4. Baseline characteristics for patients dropping out of the study.

Selected baseline characteristics resulting from the primary analysis contrasted by the patients who dropped out of the study post-baseline. Data are *n* (%) or mean±standard deviation unless otherwise stated.

	Primary analysis population		Population dropping out†	
	Linagliptin (*n*=1618)	Glimepiride (*n*=1545)	Linagliptin (*n*=267)	Glimepiride (*n*=299)
Male/Female	1002 (61.9)/ 616 (38.1)	958 (62.0)/ 587 (38.0)	167 (62.5)/ 100 (37.5)	183 (61.2)/116 (38.8)
Age, years	64.4±9.1	64.4±9.3	64.0±10.6	65.0±10.2
History of myocardial infarction	266 (14.0)	187 (12.1)	39 (14.6)	51 (17.1)
History of cerebrovascular disease	169 (10.4)	154 (10.0)	31 (11.6)	39 (13.0)
Known coronary artery disease	394 (24.4)	366 (23.7)	66 (24.7)	95 (31.8)
Education level, years	10.8±3.4	10.8±3.5	10.9±3.7	10.9±3.4
Body Mass Index, kg/m^2	30.8±5.0	30.7±4.9	30.9±5.6	30.8±5.4
Mini-mental state examination score	28.5±1.7	28.5±1.7	28.3±1.7	28.2±1.8
Depression score according to CES-D	8.7±8.0	9.3±8.3	9.7±8.8	10.6±9.3
<16	1335 (82.5)	1242 (80.4)	206 (77.2)	223 (74.6)
≥16	250 (15.5)	278 (18.0)	55 (20.6)	63 (21.1)
Missing	33 (2.0)	25 (1.6)	6 (2.2)	13 (4.3)
eGFR (MDRD), ml/min/1.73 m^2	75.8±19.0	76.9±18.8	75.4±19.6	75.2±19.7
Type 2 diabetes duration, years	7.7±6.2	7.4±5.9	8.1±6.6	8.0±6.5
HbA1c, mmol/mol (%)	54.3±6.0 (7.1±0.5)	54.5±6.2 (7.1±0.6)	55.3±6.4 (7.2±0.6)	54.8±6.2 (7.2±0.6)
Glucose-lowering medication				
Metformin	1348 (83.3)	1306 (84.5)	231 (86.5)	257 (86.0)
Sulphonylurea	434 (26.8)	422 (27.3)	61 (22.8)	75 (25.1)
Glinide	13 (0.8)	13 (0.8)	1 (0.4)	2 (0.7)
α-glucosidase inhibitor	43 (2.7)	34 (2.2)	2 (0.7)	10 (3.3)
Thiazolidinedione	1 (0.1)	2 (0.1)	0 (0.0)	0 (0.0)
Insulin use	1 (0.1)	0 (0.0)	0 (0.0)	0 (0.0)

Cardiovascular medications				
Lipid-lowering	1197 (74.0)	1180 (76.4)	187 (70.0)	221 (73.9)
Statins	1111 (68.7)	1113 (72.0)	175 (65.5)	206 (68.9)
Antihypertensives	1428 (88.3)	1387 (89.8)	240 (89.9)	272 (91.0)
Systolic blood pressure, mmHg	135.9±15.9	136.2±16.4	136.0±16.6	136.8±16.1
Diastolic blood pressure, mmHg	78.8±9.5	78.8±9.3	78.6±9.8	79.0±9.8
LDL cholesterol, mmol/L	2.4±0.9	2.4±0.9	2.5±0.9	2.5±0.9

[†]See ESM Figure 2: participants dropped-out between ③ and ④ i.e. participants without a post-baseline RBI assessment. Participants with a post-baseline assessment that was not on treatment (n=289; i.e. between ④and ⑤) are considered in sensitivity analysis 5 (ESM Table 3). CES-D: Center for Epidemiologic Studies Depression Scale. eGFR: estimated glomerular filtration rate, LDL: low-density lipoprotein, MDRD: Modification of Diet in Renal Disease study equation.

ESM Table 5. Changes in MMSE, A&E z-score, TMT and VFT by accelerated cognitive decline (yes/no) regardless of treatment group at week 160 and end of follow-up.

	Accelerated cognitive decline at week 160*		Accelerated cognitive decline at end of follow-up	
RBI at or below 16th percentile†				
MMSE score	Yes	No	Yes	No
Follow-up	26.2 (2.7)	29.0 (1.2)	25.7 (3.7)	29.0 (1.2)
n	827	2220	628	1687
Change from baseline	-1.9 (2.5)	0.4 (1.4)	-2.5 (3.6)	0.4 (1.6)
n	827	2220	628	1687
A&E z-score	Yes	No	Yes	No
Follow-up	-0.7 (0.8)	0.2 (0.5)	-0.7 (0.8)	0.1 (0.6)
n	812	2151	606	1674
Change from baseline	-0.6 (0.8)	0.1 (0.6)	-0.7 (1.0)	0.1 (0.7)
n	800	2119	596	1648
TMT A score [in sec]	Yes	No	Yes	No
Follow-up	62.9 (42.3)	50.1 (24.0)	63.7 (43.7)	51.6 (25.9)
n	728	2013	559	1589
Change from baseline	4.4 (34.0)	1.7 (20.4)	6.3 (37.2)	3.1 (22.1)
n	693	1918	524	1509
TMT B score [in sec]	Yes	No	Yes	No
Follow-up	164.9 (79.3)	101.5 (47.5)	170.1 (81.5)	110.1 (53.0)
n	727	2016	545	1585
Change from baseline	38.9 (71.7)	-2.4 (41.1)	47.4 (78.9)	6.5 (50.4)
n	665	1881	490	1479
TMT ratio	Yes	No	Yes	No
Follow-up	2.3 (1.4)	1.1 (0.6)	2.4 (1.5)	1.2 (0.7)
n	711	1994	534	1573
Change from baseline	0.9 (1.4)	-0.1 (0.8)	1.0 (1.6)	-0.01 (0.9)
n	642	1836	471	1445

VFT FAS score	Yes	No	Yes	No
Follow-up	7.4 (3.6)	9.6 (3.9)	7.1 (3.8)	9.8 (4.4)
n	753	2032	562	1566
Change from baseline	-0.9 (2.6)	0.04 (2.6)	-1.3 (3.2)	0.2 (3.1)
n	748	2019	557	1556
VFT animals score	Yes	No	Yes	No
Follow-up	12.8 (5.5)	16.6 (6.6)	12.4 (6.2)	16.8 (7.4)
n	740	1998	548	1518
Change from baseline	-1.9 (5.4)	0.1 (5.9)	-2.5 (6.5)	0.1 (7.1)
n	731	1963	535	1494
RBI at or below 10th percentile[‡]				
MMSE score	Yes	No	Yes	No
Follow-up	25.7±2.9	28.8±1.4	25.0±4.2	28.8±1.5
n	539	2508	411	1904
Change from baseline	-2.4±2.7	0.2±1.5	-3.1±4.1	0.2±1.7
n	539	2508	411	1904
A&E z-score	Yes	No	Yes	No
Follow-up	-0.8±0.8	0.1±0.6	-0.9±0.9	0.1±0.6
n	526	2437	394	1886
Change from baseline	-0.7±0.9	0.1±0.6	-0.8±1.0	0.02±0.68
n	520	2399	387	1857

*Data on cognitive change in the first time window are based on actual or interpolated cognitive values (ESM Figure 1, scenario 1, 2 and 3); data from all participants in the primary analyses are represented in this time window, but numbers per test vary according to test availability at both baseline and follow-up. Because A&E z-scores, VFT and TMT scores could be missing at baseline, *n* for follow-up scores can be higher than *n* for calculated change from baseline.

Data on cognitive change in the second time window only involve participants with an actual assessment in that window (ESM Figure 1, scenario 1 and 3); data from participants with an end of treatment assessment only in time window 1 (who are included in the primary analysis) are not included in these averages.

MMSE: Mini-Mental State Examination, A&E: attention and executive functioning, TMT: Trail Making Test, VFT: Verbal Fluency Test, RBI: regression-based index score. [‡†]: RBI 10th/16th percentile thresholds: at week 160 for MMSE z-score:-1.3388/-0.8562; at end of follow-up for MMSE z-score: -1.0927/-0.7218; at week 160 for A&E z-score:-1.2281/-0.8640; at end of follow-up for A&E z-score: -1.1723/-0.8839.

ESM Table 6. Results of the primary and secondary analyses for accelerated cognitive decline (ACD).

	Linagliptin (*n* = 1618) *n* (%)	Glimepiride (*n* = 1545) *n* (%)	Comparison OR [95% CI][1]	*p*- value
Primary endpoint:				
Incidence of ACD at EOT based on 16th percentile of RBI score	449 (27.8)	426 (27.6)	1.01 [0.86, 1.18]	0.91
Secondary and further endpoints :				
Incidence of ACD at week 160 based on RBI score.	446 (27.6)	406 (26.3)	1.07 [0.91, 1.25]	0.41
Incidence of ACD based on z-score at EOT[2]	537 (33.2)	509 (32.9)	1.01 [0.87, 1.17]	0.88
Incidence of ACD based on z-score at week 160[2]	498 (30.8)	467 (30.2)	1.03 [0.88, 1.19]	0.74
Incidence of ACD at EOT based on 10th percentile of RBI Score[3]	296 (18.3)	291 (18.8)	0.96 [0.81, 1.15]	0.70
Incidence of ACD at week 160 based on 10th percentile of RBI score[3]	295 (18.2)	263 (17.0)	1.09 [0.91, 1.31]	0.37
Incidence of ACD at EOT based on MMSE[4]	116 (7.2)	123 (8.0)	0.90 [0.69, 1.17]	0.41
Incidence of ACD at week 160 based on MMSE[4]	98 (6.1)	93 (6.0)	1.01 [0.75, 1.35]	0.95

1: Odds Ratio (OR) along with the 95% Profile Likelihood Confidence Interval (CI) and the two-sided *p*-value using a logistic regression including treatment as a factor. 2: Incidence of accelerated cognitive decline based on 16th percentile of z-score for MMSE and/or A&E. 3: Incidence of accelerated cognitive decline based on 10th percentile of RBI score. 4: Incidence of accelerated cognitive decline at EOT based on MMSE score of <24 or a decline of >4 points in MMSE score at EOT relative to baseline ACD: cognitive accelerated decline.

The R^2‘s obtained from the ANCOVA (see ESM text methods 1) were 25.4% and 16.5%, respectively for the predicted MMSE scores at 160 weeks and at EOT. The corresponding R^2‘s were 24.5% and 18.9% for the predicted A&E z-scores.

ESM Table 7. Changes in cognitive or depression scores after 160 weeks of follow-up.

	Linagliptin (*n*=1618)	Glimepiride (*n*=1545)	Comparison	*p*-value
Changes (Δ) from baseline in raw or z-scores	Adjusted mean (SE)	Adjusted mean (SE)	Adjusted mean (SE) [95% CI]	
Δ MMSE score	−0.24 (0.049)	−0.23 (0.050)	−0.01 (0.070) [−0.14, 0.13]	0.9343
Δ MMSE z-score	−0.14 (0.029)	−0.14 (0.030)	~0.00 (0.042) [−0.08, 0.08]	0.9343
Δ A&E z-score	−0.05 (0.016)	−0.06 (0.017)	0.01 (0.024) [−0.04, 0.05]	0.8108
Δ TMT A score	2.33 (0.640)	2.79 (0.658)	−0.46 (0.918) [−2.26, 1.34]	0.6135
Δ TMT A z-score	0.08 (0.023)	0.10 (0.024)	−0.02 (0.033) [−0.08, 0.05]	0.6135
Δ TMT B score	7.95 (1.400)	9.92 (1.431)	−1.96 (2.002) [−5.89, 1.96]	0.3269
Δ TMT B z-score	0.13 (0.023)	0.16 (0.024)	−0.03 (0.033) [−0.10, 0.03]	0.3270
Δ TMT ratio score	0.13 (0.027)	0.16 (0.028)	−0.04 (0.039) [−0.11, 0.04)	0.3364
Δ TMT ratio z-score	0.12 (0.026)	0.16 (0.027)	−0.04 (0.038) [−0.11, 0.04]	0.3364
Δ VFT animals -15 seconds z-score	~0.00 (0.024)	−0.04 (0.024)	0.04 (0.034) [−0.03, 0.11]	0.2238
Δ VFT FAS -15 seconds z-score	−0.05 (0.018)	−0.02 (0.018)	−0.02 (0.026) [−0.07, 0.03]	0.3321
Δ VFT animals - 60 seconds z-score	−0.08 (0.022)	−0.07 (0.023)	~0.00 (0.032) [−0.07, 0.06]	0.8919
Δ VFT FAS - 60 seconds z-score	−0.06 (0.016)	−0.05 (0.016)	−0.01 (0.023) [−0.05, 0.04]	0.6775
Δ VFT overall - 15 seconds z-score	−0.02 (0.017)	−0.03 (0.018)	0.01 (0.025) [−0.04, 0.06]	0.6860
Δ VFT overall - 60 seconds z-score	−0.07 (0.016)	−0.07 (0.016)	~0.00 (0.022) [−0.05, 0.04]	0.9664
Δ CES-D score	1.60 (0.239)	2.29 (0.246)	−0.69 (0.343) [−1.36, −0.02]	0.0448

For comparison: adjusted means (SE) along with the 95% Confidence Interval (CI) and the two-sided *p*-value are shown using an MMRM model including treatment, week and baseline value, treatment by week interaction and baseline by week interaction as covariates. MMSE: Mini-Mental State Examination VFT: Verbal Fluency Test TMT: Trail Making Test TMT ratio: (TMT-B – TMT-A)/TMT-A, CES-D: Center for Epidemiologic Studies Depression Scale, ACD: accelerated cognitive decline

ESM table 8. Changes in cognitive or depression scores at end of follow-up.

	Linagliptin (*n* = 1618)	Glimepiride (*n* = 1545)	Comparison	*p*- value
Changes (Δ) from baseline in raw or z-scores	Adjusted mean (SE)	Adjusted mean (SE)	Adjusted mean (SE) [95% CI]	
Δ MMSE score	−0.42 (0.071)	−0.47 (0.074)	0.05 (0.103) [−0.15, 0.25]	0.6383
Δ MMSE z-score	−0.25 (0.042)	−0.28 (0.044)	0.03 (0.061) [−0.09, 0.15]	0.6383
Δ A&E z-score	−0.11 (0.020)	−0.12 (0.021)	0.01 (0.029) [−0.05, 0.07]	0.7721
Δ TMT A score	3.48 (0.770)	4.35 (0.799)	−0.87 (1.110) [−3.05, 1.31]	0.4335
Δ TMT A z-score	0.13 (0.028)	0.16 (0.029)	−0.03 (0.040) [−0.11, 0.05]	0.4336
Δ TMT B score	16.52 (1.749)	18.11 (1.804)	−1.59 (2.513) [−6.52, 3.34]	0.5264
Δ TMT B z-score	0.27 (0.029)	0.30 (0.030)	−0.03 (0.041) [−0.11, 0.06]	0.5265
Δ TMT ratio score	0.25 (0.032)	0.24 (0.034)	0.02 (0.047) [−0.08, 0.11]	0.7332
Δ TMT ratio z-score	0.25 (0.032)	0.23 (0.033)	0.02 (0.046) [−0.07, 0.11]	0.7332
Δ VFT animals -15 seconds z-score	0.03 (0.031)	0.04 (0.032)	−0.01 (0.045) [−0.09, 0.08]	0.8941
Δ VFT FAS -15 seconds z-score	−0.01 (0.023)	~0.00 (0.023)	−0.01 (0.033) [−0.07, 0.06]	0.8049
Δ VFT animals - 60 seconds z-score	−0.09 (0.030)	−0.11 (0.031)	0.03 (0.044) [−0.06, 0.11]	0.5605
Δ VFT FAS - 60 seconds z-score	−0.05 (0.021)	−0.05 (0.022)	~0.00 (0.031) [−0.06, 0.06]	0.9814
Δ VFT overall - 15 seconds z-score	0.01 (0.023)	0.02 (0.024)	~0.00 (0.033) [−0.07, 0.06]	0.8835
Δ VFT overall - 60 seconds z-score	−0.07 (0.021)	−0.09 (0.022)	0.02 (0.031) [−0.04, 0.08]	0.5482
Δ CES-D score	1.46 (0.259)	1.54 (0.268)	−0.07 (0.373) [−0.80, 0.66]	0.8443

For comparison: adjusted means (SE) along with the 95% Confidence Interval (CI) and the two-sided *p*-value are shown using an MMRM model including treatment, week and baseline value, treatment by week interaction and baseline by week interaction as covariates. MMSE: Mini-Mental State Examination, VFT: Verbal Fluency Test, TMT: Trail Making Test, TMT ratio: (TMT-B – TMT-A)/TMT-A, CES-D: Center for Epidemiologic Studies Depression Scale, ACD: accelerated cognitive decline

ESM Figure 1. Handling of missing cognitive assessments for the primary cognitive outcome (occurrence of ACD at end of follow-up and at week 160).

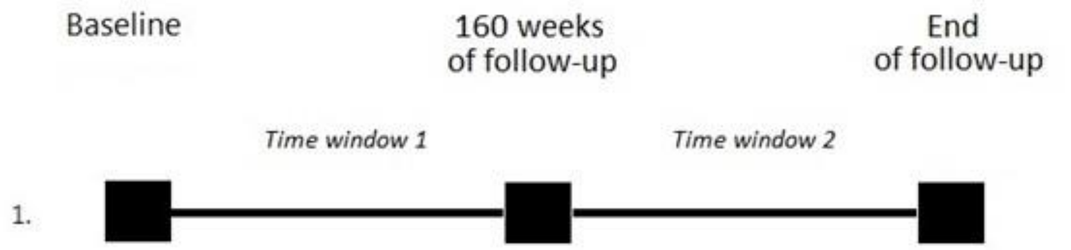

Subject with assessment in the first and second time window. The first assessment was planned at 160 weeks. The second assessment (end of follow-up) was the final assessment of the subject in time window 2, either at termination of the trial, or at earlier individual close-out.
Linagliptin: *n* = 1165, Glimepiride: *n* = 1072, total: *n* = 2237

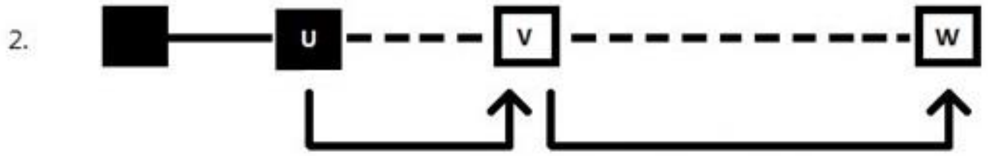

Subject with only a close-out assessment (U) in the first time window prior to the planned assessment at 160 weeks (V). The classification for ACD (yes/no) at V was based on the assessment U, taking into account duration of follow-up. The classification for ACD (yes/no) at W, end of follow-up and primary cognitive outcome measure, was identical to the classification at V.
Linagliptin: *n* = 410, Glimepiride: *n* = 425, total: *n* = 835

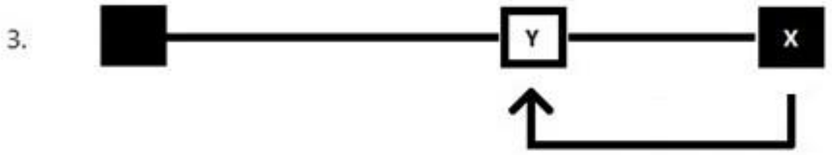

Subject with only an assessment in the second time window (X). The missing values at Y was interpolated based on measurements at baseline and at X for each available cognitive test. ACD at 160 weeks will be based on RBI calculated for interpolated test scores at Y.
Linagliptin: *n* = 43, Glimepiride: *n* = 48, total: *n* = 91

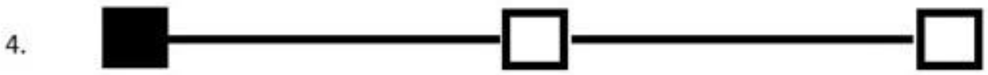

Subject without an on-treatment assessment. This subject was excluded from the primary analysis.

ESM Figure 2. CAROLINA-COGNITION study flow-chart.

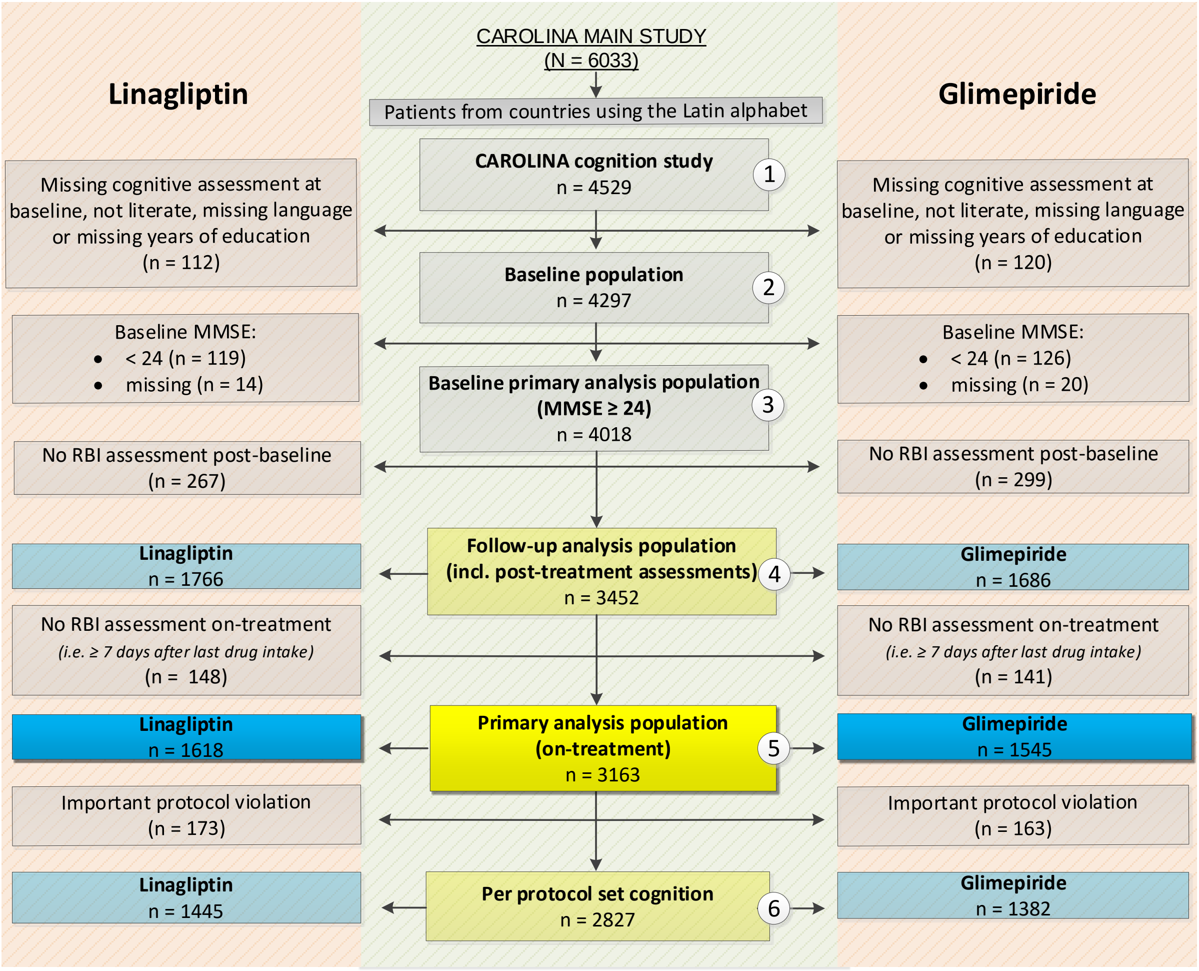

① Includes all participants from countries using the Latin alphabet who were dispensed study medication and were documented to have taken at least one dose of investigational treatment (treated set).

② Includes all participants in ① who have baseline assessment (of which at least one of the z-scores, A&E or MMSE can be calculated), are literate and their years of formal education are available (BL-COG).

③ All participants in ② with MMSE ≥24 at baseline. This set is used for language correction and calculation of z-scores (BL-COG-ELIG).

④ Includes all participants included in ③ who have at least one post-baseline assessment (on-treatment, or post-treatment, FAS-COG-EXT). Used for sensitivity analysis 5 in ESM Table 3.

⑤ Includes all participants included in ③ who have at least one on-treatment assessment or who had a valid baseline assessment but did not understand the test instructions at the subsequent assessment and were therefore classified as having ACD (i.e. within 7 days after treatment stop) ; this set is used for the primary analysis (FAS-COG).

⑥ Includes all participants in ⑤ who do not have an important protocol violation (i.e. psychiatric disease or history of alcohol/drug abuse prior to inclusion; PPS-COG). Used for sensitivity analysis 1 in ESM Table 3.

A&E: attention and executive functioning, MMSE: Mini-Mental State Examination, RBI: regression based index.

ESM Figure 3. Participating countries and number of participants per country in the CAROLINA-COGNITION study.

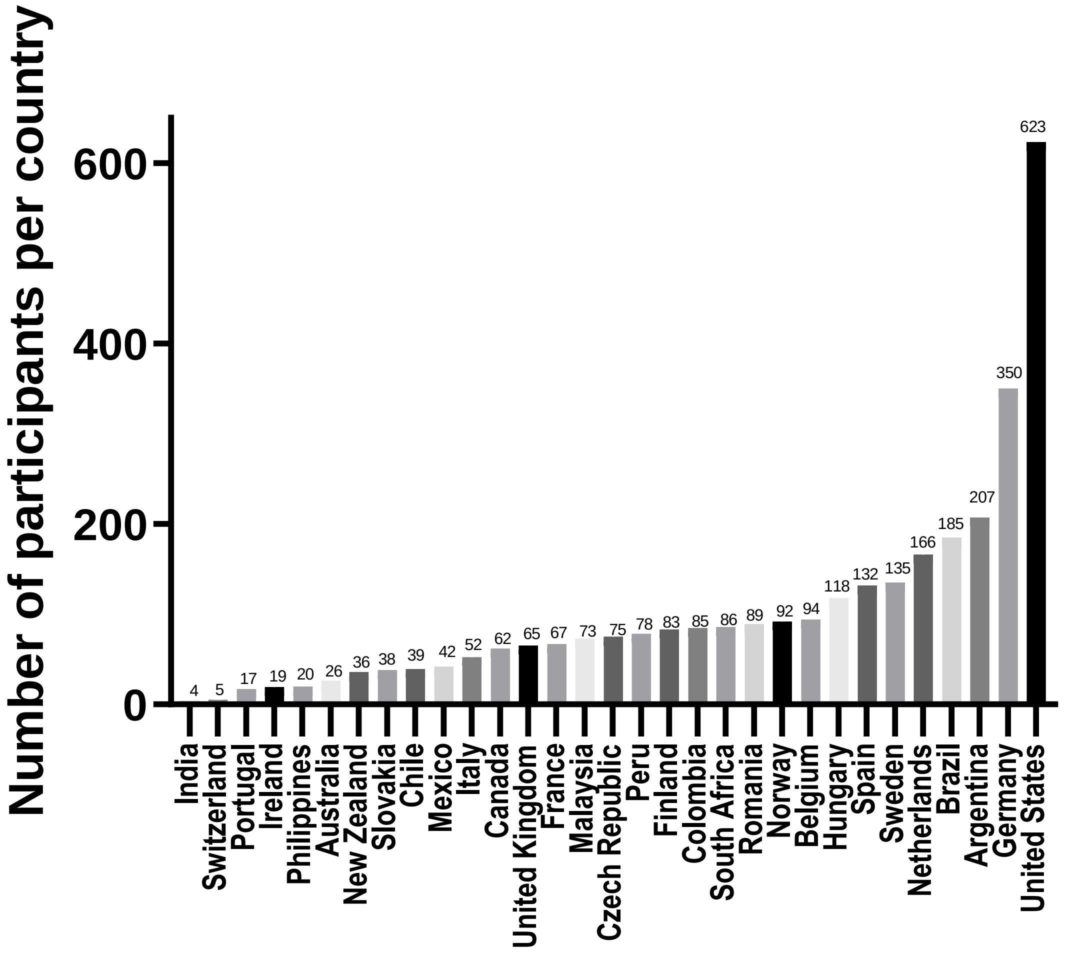

ESM figure 4. HbA1c over time in participants in the CAROLINA-COGNITION study.

A. Mmol/mol-change

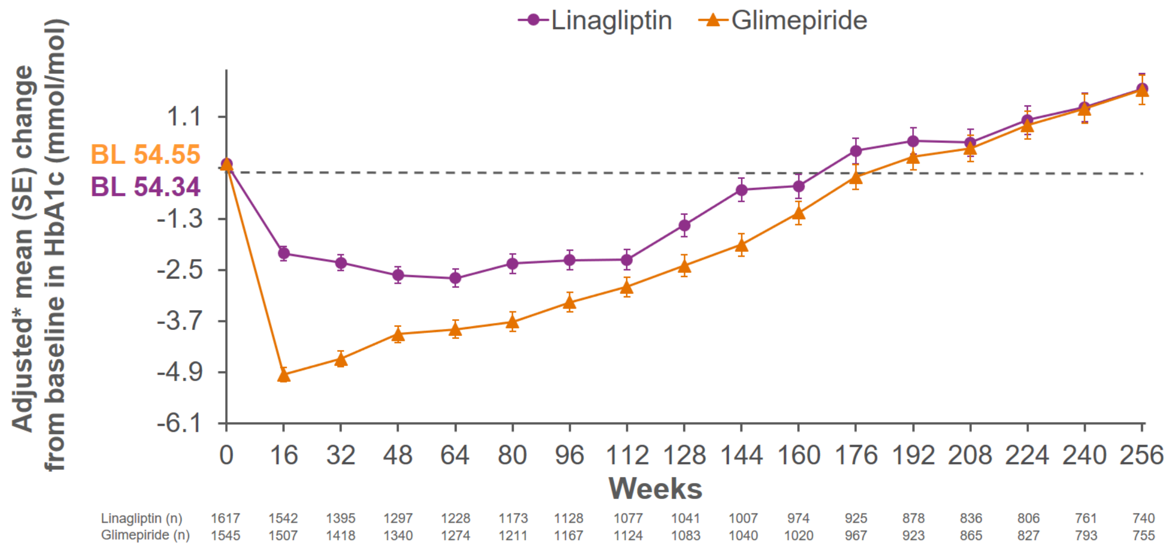

OC analysis. *Based on MMRM including treatment, week repeated within participants, week by treatment interaction, continuous baseline HbA1c and baseline HbA1c by week interaction. BL, baseline; MMRM, mixed-model repeated measures

B. %-change

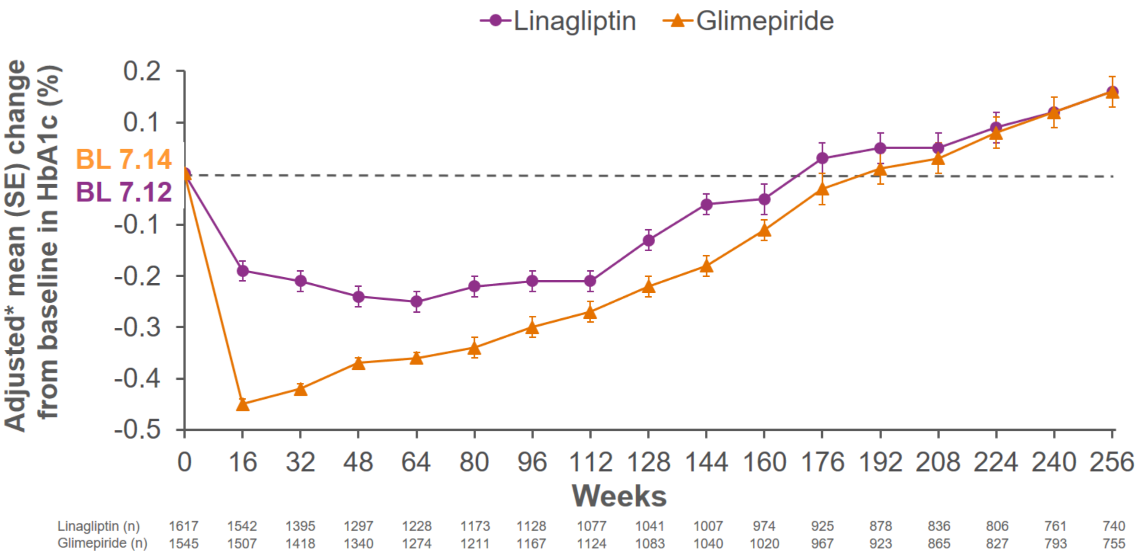

OC analysis. *Based on MMRM including treatment, week repeated within participants, week by treatment interaction, continuous baseline HbA1c and baseline HbA1c by week interaction. BL, baseline; MMRM, mixed-model repeated measures

ESM figure 5. Weight over time in participants in the CAROLINA-COGNITION study.

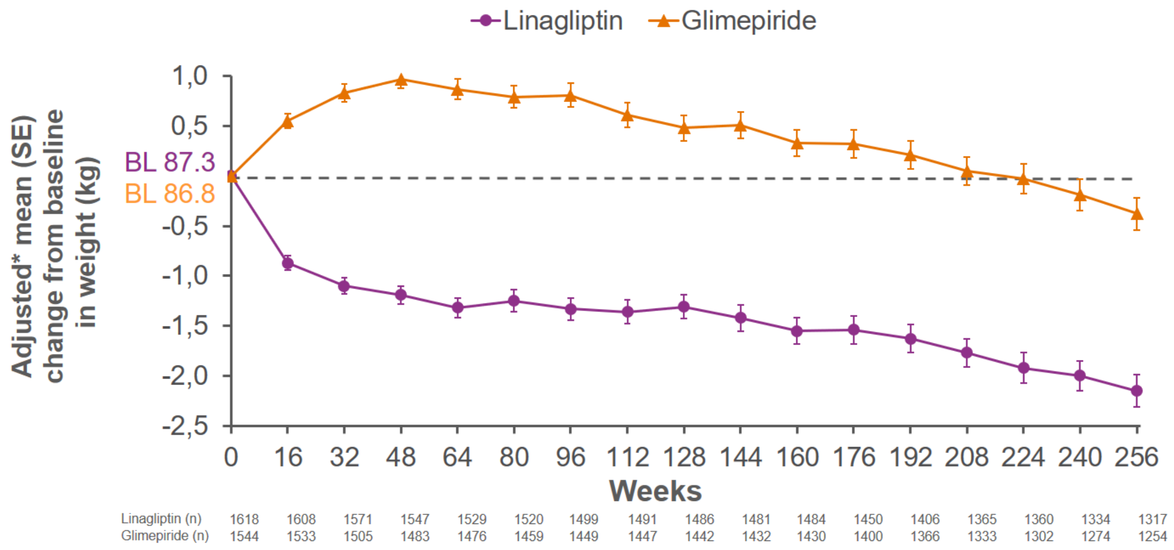

OC-ROC. *Based on MMRM including treatment, week repeated within participants, week by treatment interaction, continuous baseline weight and baseline weight by week interaction. BL, baseline; MMRM, mixed-model repeated measures

ESM figure 6. Proportion of participants in the CAROLINA-COGNITION study with hypoglycaemia.

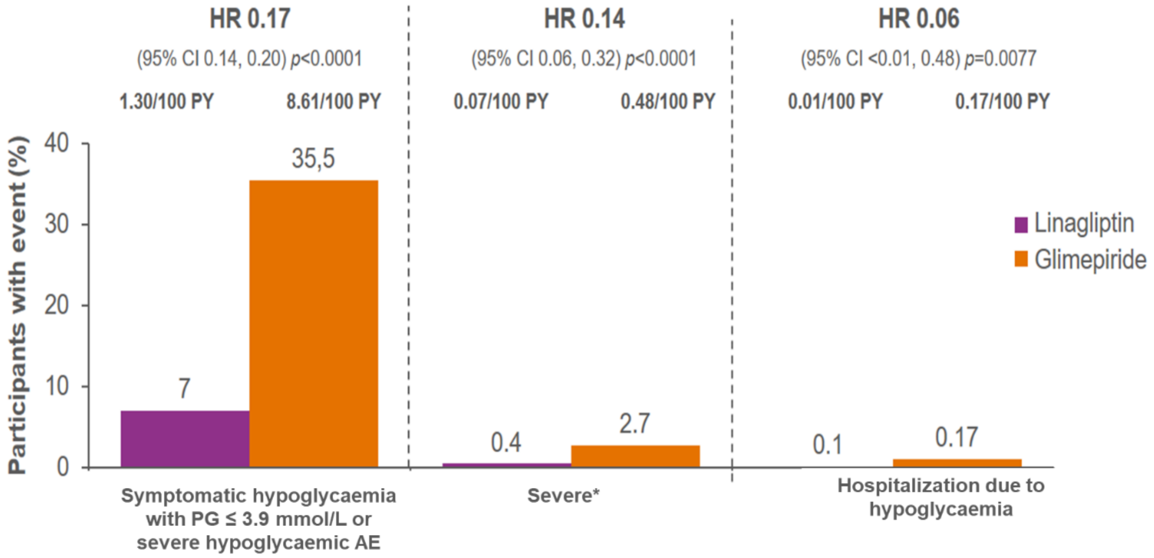

Events occurring between first study drug intake until 7 days after last permanent study drug stop. *Hypoglycaemic event requiring the assistance of another person to actively administer carbohydrate, glucagon or other resuscitative actions. AE – adverse event.
